# Usefulness and applicability of web-based headache diagnostic software

**DOI:** 10.1186/1129-2377-14-S1-P142

**Published:** 2013-02-21

**Authors:** A Radojicic, M Nikolic, J Zidverc-Trajkovic, A Podgorac, N Sternic

**Affiliations:** 1 Headache Center, Neurology Clinic, Clinical Center of Serbia, Serbia and Montenegro; 2 Faculty of Mathematics, University of Belgrade, Serbia, Serbia and Montenegro

## Introduction

Headache diagnostic software (HDS), developed by Headache center, Neurology Clinic in Belgrade integrates a diagnostic expert system able to suggest the correct ICHD-II diagnosis once all clinical characteristics of a patient’s headache have been collected.

Purpose The aim of this pilot study was to test its diagnostic accuracy, the usefulness and applicability in the diagnosis of primary headache disorders and medication overuse headache (MOH).

## Methods

Forty patients with clinical diagnosis of migraine, tension type headache (TTH), cluster headache and MOH with an Internet access agreed to fill the web-based headache diary 4-6 weeks after their first consultation in Headache center. This diagnostic diary is developed exclusively based on the ICHD-II criteria, and represents the core of HDS. We analyzed the level of agreement between diagnosis given after clinical interview and obtained by HDS as well as patients’ level of compliance.

## Results

Patients’ understanding of the diary proved highly satisfactory which was followed by good compliance (77.5%). Number of patients with one headache diagnosis was 22; 9 patients received 2 or 3 different diagnosis. All patients with a clinical diagnosis of migraine (21) had at least one migraine attack recognized by HDS; episodic TTH was less frequent diagnosed using standard clinical interview than by DHS. In all MOH (4) and cluster headache patients (3) there was complete concordance between clinical and software generated diagnosis.

## Conclusions

Web-based HDS can be considered as advanced, user-friendly, useful and reliable diagnostic tool in headache center.
